# Nonrenewable Energy Cost and Greenhouse Gas Emissions of a “Pig-Biogas-Fish” System in China

**DOI:** 10.1100/2012/862021

**Published:** 2012-11-08

**Authors:** Qing Yang, Xiaofang Wu, Haiping Yang, Shihong Zhang, Hanping Chen

**Affiliations:** ^1^Department of New Energy Science and Engineering, School of Energy and Power Engineering, Huazhong University of Science & Technology, Wuhan 430074, China; ^2^State Key Laboratory of Turbulence and Complex Systems, College of Engineering, Peking University, Beijing 100871, China; ^3^State Key Laboratory of Coal Combustion, Huazhong University of Science & Technology, Wuhan 430074, China

## Abstract

The purpose of this study is to assess the energy savings and emission reductions of the present rural biogas system in China. The life cycle assessment (LCA) method is used to analyze a “pig-biogas-fish” system in Jingzhou, Hubei Province, China. The nonrenewable energy cost and the greenhouse gas (GHG) emissions of the system, including the pigsty, the biogas digester, and the fishpond, are taken into account. The border definition is standardized because of the utilization of the database in this paper. The results indicate that the nonrenewable energy consumption intensity of the “pig-biogas-fish” system is 0.60 MJ/MJ and the equivalent CO_2_ emission intensity is 0.05 kg CO_2_-eq/MJ. Compared with the conventional animal husbandry system, the “pig-biogas-fish” system shows high renewability and GHG reduction benefit, which indicates that the system is a scientific and environmentally friendly chain combining energy and ecology.

## 1. Introduction

Nowadays resources and environment have been the two focus of attention [1–3]. As a developing country, 60% population of which are peasants, China always takes development of economy and ecology of the rural area as one of the most important works [4]. To propel the sustainable development of the rural economy and to promote the continuous improvement of ecological environment, the Chinese government has long promoted biogas construction and has given it policy preferences, financial support, and technology inputs [5]. However, the biogas system is an energy conversion process, which will necessarily consume nonrenewable energy and discharge greenhouse gas (GHG) [6]. So it is meaningful to study the present rural biogas system over its entire life cycle. 

The International Standardization Organization (ISO) defines life cycle assessment (LCA) as the following: “compilation and evaluation of the inputs, outputs and the potential environmental impacts of a product system throughout its life cycle” [7]. Based on research experiences of other scholars and from our early studies [8–12], LCA can offer a comprehensive way to assess the energy consumption and greenhouse gas emissions of the given systems. 

**Figure 1:**
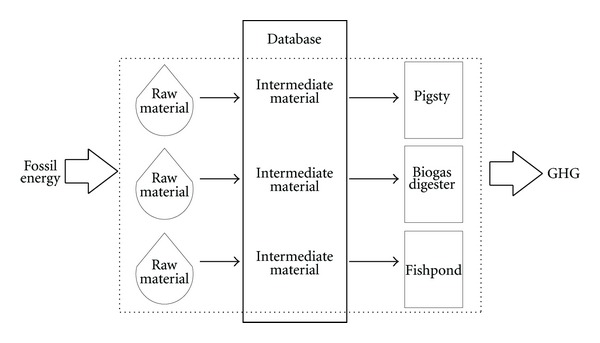
The boundary of the “pig-biogas-fish” system.

Several researchers have analyzed typical biogas systems using the LCA method. Some focused on the biogas technologies designed in laboratory [13, 14], and some focused on the biogas engineering itself [15, 16]. Patterson et al. [17] provided an assessment of biogas systems on a regional scale in the UK that can provide guidance on infrastructure development decisions; Martin et al. [18] utilized a life cycle approach to present the environmental impacts of the integration of biogas and ethanol processes; Wei et al. [19] assessed the efficiency and sustainability of the “Four in One” ecological economic system for peach production system in Beijing by life cycle energy analysis; Wang et al. [20] calculated and evaluated the energy conservation and the emission reductions of the rural household biogas project in China by establishing the LCA method. In these previous researches, when setting the system boundary, human factors play a significant role. For a system, different researchers may get absolutely different results because of different boundary definition. For example, some researchers take transportation processes into account [21, 22], while some others do not [23], so the comparability of their data disappears. In this paper, the Chinese National Economy System Ecological Elements Database established by Zhou [24] is used for the calculation of the relevant ecological elements. Based on the system input and output of the simulation method, the Chinese National Economy System Ecological Elements Database is built in view of energy consumption, greenhouse gas emissions, and other key factors affecting the environment. Because of the certainty of the defining of the boundary in the database, the border definition is simplified and standardized. LCA is used to analyze a chosen rural household “pig-biogas-fish” system in the Zhongzhouzi fishery, Jingzhou Hubei Province in this research. Besides the biogas link, the upstream pigsty link, and, the downstream fishpond link are taken into account as a system, as showed in Figure 1. Also, the nonrenewable energy cost and the GHG emissions of this total system are calculated and compared to those of the conventional animal husbandry system.

## 2. Materials and Methods

### 2.1. Model of the “Pig-Biogas-Fish” System

**Figure 2:**
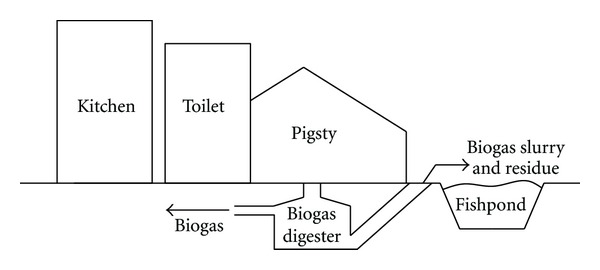
A schematic diagram of the “pig-biogas-fish” system.

The “pig-biogas-fish” system is a key unit to combine clean energy production and animal husbandry in China [25], and it works as follows: through raising pigs, farmers put the pig manure into the digester as the fermentation crude to product biogas for everyday lighting and cooking. Meanwhile, the biogas slurry and residue can be used as a base fertilizer and top dressing for the fishpond, as showed in Figure 2. The data of the “pig-biogas-fish” system in this study is provided by the survey of the Zhongzhouzi fishery, which is organized by the authors. The “pig-biogas-fish” system covers an area of about 5320 m^2^, and it is designed with an operational life of 20 years. Below the elements of each link are described and analyzed separately, and the main consideration is the productions in the construction, the operation, and the maintenance phases. One year is chosen as the time span for this study.

The pigsty covers an area of 20 m^2^, and its construction investment is 2,000 Yuan, including cement, lime, hollow bricks, steel, and so forth. The main consumption in the daily operation of the pigsty is feed, vaccines, insect repellent, medicine, and disinfectant. The statistics show that on average a pig needs 363 kg of feed to grow to 100 kg, and each year it consumes about 6 g of drugs, such as the vaccine and the insect repellent. The pigsty needs to be disinfected at regular intervals.

This system includes an 8 m^3^ biogas pool, with a cylindrical type. Its area is not considered. Its construction materials consist of 500 grade cement, fine sand, pebble, and a plastic discharge pipe 16 cm in diameter and 1.8 m in length, a plastic discharge pipe 20 cm in diameter and 0.8 m in length, a feed pipe 22 cm in diameter and 1.2 m in length, and an 8 m^3^ steel mold.

The fishpond is excavated on the base of a natural small lake, covering an area of 5300 m^2^. The main consideration is the investment in the fishpond operation and maintenance phases, and the investment in construction is ignored in this study. In the fish farming process, lime is needed regularly to disinfect the pond and bleach is used to prevent fish diseases. Beside biogas manure, nitrogen and phosphate fertilizer is applied to the fishpond for promoting the growth of aquatic plants. A certain amount of concentrated feed is also needed to ensure production.

The pigsty in this “pig-biogas-fish” system has an annual output of 8 pigs, with an average of 125 kg per head. This system produces 400 m^3^ of biogas each year. The annual output of the fishpond is 2 kg/m^2^.  Table 1 is the statistical result of the energy content in the outputs for the system.

### 2.2. Model of the Conventional Animal Husbandry System

The conventional animal husbandry system consists of a pigsty with an area of 20 m^2^ and a fishpond covering an area of 5300 m^2^. In this paper, the model of the conventional animal husbandry system is set up based on the “pig-biogas-fish” system introduced above. The conventional animal husbandry system covers an area of about 5320 m^2^, and its operational life is calculated as 20 years. In the conventional animal husbandry system, coal is used for everyday lighting and cooking, the energy of which is equal to the energy of the biogas produced in the “pig-biogas-fish” system. Without biogas manure, the qualities of the nitrogen and phosphate fertilizer applied to the fishpond in the conventional system, respectively, are 9 times more than those of the “pig-biogas-fish” system. The detailed inventories of the two systems are analyzed later in this paper.

### 2.3. Nonrenewable Energy Cost

To analyze the nonrenewable energy consumption of the system, we use the life cycle embodied energy method, an important type of the energy analysis methods [29–32]. Reister [33] has proposed energy intensity to quantify the energy embodied in goods, similar to energy conversion rate. However, his concept does not identify the renewable energy compound and the nonrenewable energy compound of the energy consumption. Therefore, FE is defined in this paper to show how much nonrenewable energy is used directly and indirectly in the whole process, including the system establishment, operation, and maintenance. And FE can be calculated as

(1) FE=∑FEi=∑Inputi×Ci,

where FE_*i*_ denotes the nonrenewable energy used directly and indirectly in the production of the *i*th input, Input_*i*_, to the entire process of the biogas system. To calculate the proportion of the unit primary nonrenewable energy used directly and indirectly in the production or preparation of the *i*th input, *C*_*i*_ is defined as the nonrenewable energy intensity coefficient of the *i*th input. Such coefficients in this research are valued based on the Chinese National Economy System Ecological Elements Database. Therefore, this formula can calculate the nonrenewable energy cost implicit in the background of the system.

In order to quantify and evaluate the renewability of the system, it is appropriate to use nonrenewable energy investment in energy delivered (FEIED) [34, 35] as demonstrated below:

(2) FEIED=FEEout,

where *E*_out_ is the energy content of the outputs of the system. FEIED is a proportional relationship between the nonrenewable energy consumed by the system and the nonrenewable energy replaced by the system. FEIED > 1 indicates a nonrenewable process in which more energy is consumed than energy delivered, while FEIED < 1 indicates a renewable process in which more energy is delivered than energy invested. Also, the smaller the FEIED is, the higher the renewability is.

### 2.4. GHG Emissions

 Generally the GHG emissions of a product consist of two parts. One is the direct emissions part monitored by local department, and the other is the indirect emissions' part caused by inputs during the process [36]. GHG emission intensity (EI) is defined as the amount of GHG generated by one unit output energy of the system, expressed as

(3) EI=GEEout,

where GE is the GHG emissions of the system during its entire life cycle, including the direct and indirect emissions.

In this paper, input-output (I-O) analysis and process analysis are combined to compute the GHG emissions of the “pig-biogas-fish” system. The GHG emissions linked to land use are also considered. For the “pig-biogas-fish” system, its direct GHG emissions mainly include three parts: (1)  CH_4_ released into the air by swine enteric fermentation; (2) N_2_O produced by fermentation in the biogas digester and CO_2_ generated by the biogas combustion; (3)  CO_2_ and CH_4_ released into the air by the fishpond (considered as the wetland). The direct emissions are calculated according to the statistical data. Furthermore, in the process of its construction, operation, and maintenance, the “pig-biogas-fish” system consumes some products, produced by other systems, and a certain amount of GHG is emitted during the production processes; these emissions derived from outside the biogas system are the indirect GHG emissions. Similarly, the indirect GHG emissions (GE_in_) associated with FE can be calculated as

(4) GEin=∑GEi=∑Inputi×Gi,

where GE_*i*_ denotes the GHG emissions in the production of *i*th inputs and *G*_*i*_ is defined as the GHG intensity coefficient of the *i*th inputs, valued based on the Chinese National Economy System Ecological Elements Database.

Limited to the national conditions and statistics, this study mainly considers three greenhouse gases, CO_2_, CH_4_, and N_2_O. And in accordance with the standard of 100-year scaleglobal warming potential, CH and N_2_O are equivalent to CO_2_ as 23 g/g and 296 g/g [37], respectively.

## 3. Results and Discussions

### 3.1. Calculation of the Nonrenewable Energy Cost of the “Pig-Biogas-Fish” System

**Figure 3:**
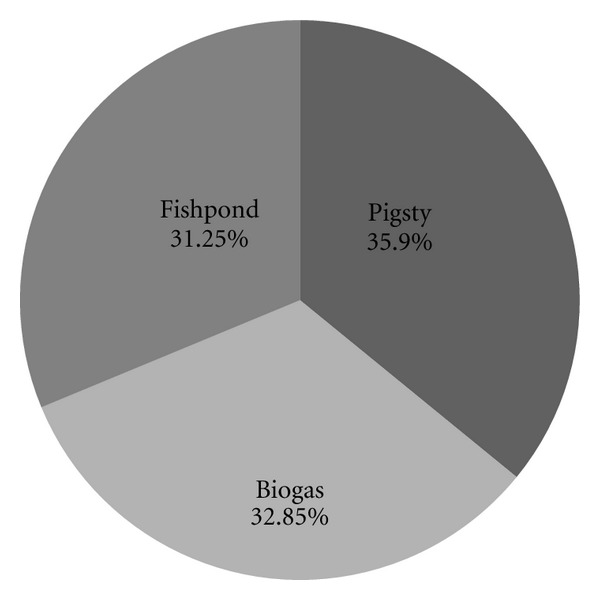
NE cost fractions for the “pig-biogas-fish” system.

The nonrenewable energy consumption of the “pig-biogas-fish” system is shown in Table 2. The total FE cost for the system is 6.80*E* + 04 MJ/yr. As listed in Table 1, the *E*_out_ of the system is 1.13*E* + 05 MJ/yr. Thus FEIED of the “pig-biogas-fish” system is evaluated as 0.60 MJ/MJ, less than 1, and it reveals that this system has renewability. Analysis of the FE cost of the system shows that the difference between the pigsty link (35.90%), the biogas link (32.85%), and the fishpond link (31.25%) is not significant (see Figure 3), and the fishpond fraction is the smallest among the three. In addition, Table 1 shows that the fishpond accounts for the largest proportion of energy outputs. So the fishpond has the highest economic benefit. This also demonstrates that the fishpond has a favorable impact on the renewability of the “pig-biogas-fish” system.

### 3.2. Calculation of the GHG Emissions of the “Pig-Biogas-Fish” System

The indirect and direct GHG emissions of the “pig-biogas-fish” system are showed separately in Tables 2 and 3, thus the total GHG emissions can be obtained (see Table 4). The total GHG emissions for the system is summed up to be 6.17*E* + 03 kg CO_2_-eq/yr. Then, EI of the “pig-biogas-fish”system is evaluated as 0.05 kg CO_2_-eq/MJ.

**Figure 4:**
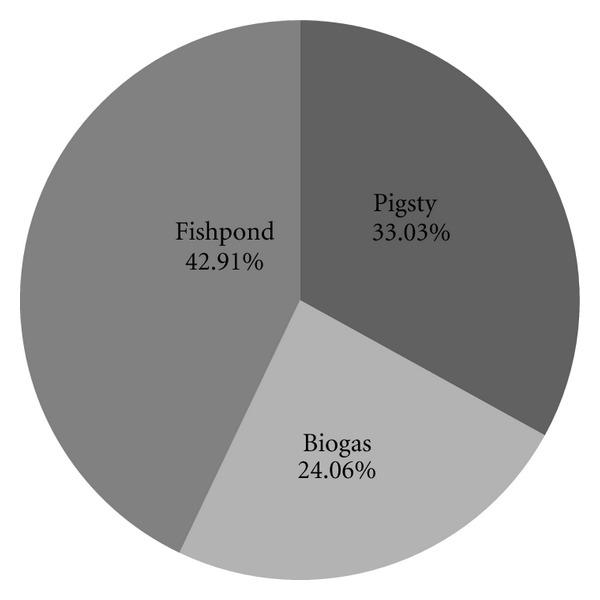
GM fractions for the “pig-biogas-fish” system.

Analysis of the GE of the system shows that the fishpond link (42.91%) is the largest contributor, followed by the pigsty link (33.03%), and the biogas link (24.06%) is the smallest one as showed in Figure 4. The GE_in_ emission inventory of the fishpond link is showed in Table 2. Because biogas manure cannot meet the need of fish farming, nitrogen and phosphate fertilizers are applied to the fishpond. These two materials account for a large proportion of the total GHG emission of the fishpond, at 16.76%. Therefore, if the nutrient content of the biogas manure could be improved by biochemical methods, the amount of these two fertilizers could be reduced and the GHG emissions would also be reduced.

### 3.3. Comparison with the Conventional Animal Husbandry System

The FE cost of the conventional animal husbandry system is 2.12*E* + 05 MJ/yr, showed in Table 5, the *E*_out_ of the system is 1.03*E* + 05 MJ/yr, and the GE of the system is summed up to be 9.59*E* + 03 kg CO_2_-eq/yr (see Table 6). Therefore, FEIED of the conventional animal husbandry system is 2.06 MJ/MJ, greater than 1, revealing that this system is a nonrenewable system, and EI of the plant is 0.09 kg CO_2_-eq/MJ. Compared with the conventional animal husbandry system, the “pig-biogas-fish” system has higher renewability because its FEIED is smaller, and the “pig-biogas-fish” system also has a higher GHG reduction benefit because its EI is smaller. This is mainly because the “pig-biogas-fish” system makes use of waste feces to provide families with the energy for everyday needs. It can therefore reduce the quantity of coal, biomass, fertilizer, and other combustions, and thus the nonrenewable energy cost and the GHG emissions are reduced, also.

At present, the national average FEIED and EI of thermal power plants are 2.64 MJ/MJ and 0.22 kg CO_2_-eq/MJ, respectively [23]. The coal power system therefore tends to consume 3.4 times more FE and 3.4 times more GHG emissions than the “pig-biogas-fish” system per unit energy output to the society.

## 4. Concluding Remarks

The system of “pig-biogas-fish” in Hubei Province, China, is analyzed by LCA in this paper. For this system, the renewability indicator FEIED, defined as nonrenewable energy investment in energy delivered, is estimated as 0.60 MJ/MJ, which shows that it has renewability. Its GHG emission intensity, EI, is calculated as 0.05 kg CO_2_-eq/MJ. Compared with the conventional animal husbandry system which consists of a pigsty and a fishpond, the “pig-biogas-fish” system has an advantage in renewability and GHG reductions.

1. FEIED of the “pig-biogas-fish” system is less than 1 and far less than that of thermal power plants in China. It indicates that the “pig-biogas-fish” system has renewability, and the fishpond link plays an important role as the analysis shows above.
2. EI of the “pig-biogas-fish” system is 1/4 of that of the present domestic coal power system, which means that as these two systems output the equal energy, the “pig-biogas-fish” system can reduce GHG emissions by 75% relative thermal power plants. Thus the rural biogas system has a positive impact on reaching the emission reduction target of China.
3. The rural biogas system, on the one hand, can meet the everyday needs of production and living for the famers, and on the other hand, it can reduce environmental pollution and make full use of biomass resources. So it is a scientific and environmentally friendly chain combining energy and ecology, in line with national conditions of China.

## Figures and Tables

**Table 1:** The output of the “pig-biogas-fish” system.

Outputs	Energy (MJ/yr)	Percentage (%)
Pig	9.80*E* + 03	8.65
Biogas	1.00*E* + 04	8.87
Fish	9.34*E* + 04	82.48

Total	1.13*E* + 05	100.00

**Table 2:** FE cost and GE_in_ emissions of the “pig-biogas-fish” system.

Links	Materials	Quantity	Unit	*C*_i_*(MJ/unit)	*G*_i_* (kg CO_2_-eq/unit)	FE (MJ/yr)	GE_in_ (kg CO_2_-eq/yr)
Pigsty	Cement	1.75*E* + 01	kg/yr	6.36	0.53	1.11*E* + 02	9.28*E* + 00
	Lime	1.25*E* + 01	kg/yr	4.94	0.79	6.18*E* + 01	9.88*E* + 00
	Hollow bricks	1.50*E* + 00	kg/yr	6.36	0.53	9.54*E* + 00	7.95*E* − 01
	Steel	2.63*E* + 00	kg/yr	64.5	2.03	1.69*E* + 02	5.33*E* + 00
	Feed	2.90*E* + 03	kg/yr	4.64	0.52	1.35*E* + 04	1.51*E* + 03
	Drugs	4.80*E* − 02	kg/yr	134	3.00	6.43*E* + 00	1.44*E* − 01
	Disinfectant	4.00*E* − 01	kg/yr	46.9	1.68	1.88*E* + 01	6.72*E* − 01
	Water	5.66*E* + 01	ton/yr	34.6	0.81	1.96*E* + 03	4.58*E* + 01
	Electricity	6.40*E* + 01	kWh/yr	135	2.82	8.64*E* + 03	1.80*E* + 02

Biogas	Cement	4.50*E* + 01	kg/yr	6.36	0.53	2.86*E* + 02	2.39*E* + 01
	Sand and pebble	3.13*E* + 00	$/yr	2.98	0.00	9.33*E* + 00	0.00*E* + 00
	Plastic pipe	4.00*E* + 00	kg/yr	108	2.99	4.32*E* + 02	1.20*E* + 01
	Steel mold	1.25*E* + 02	kg/yr	173	2.24	2.16*E* + 04	2.80*E* + 02

Fishpond	Lime	4.80*E* + 02	kg/yr	4.94	0.78	2.37*E* + 03	3.74*E* + 02
	Bleach	3.20*E* + 00	kg/yr	46.9	1.68	1.50*E* + 02	5.38*E* + 00
	Feed	2.50*E* + 02	kg/yr	4.64	0.52	1.16*E* + 03	1.30*E* + 02
	Aerator	1.25*E* + 00	$/yr	32.4	2.24	4.05*E* + 01	2.80*E* + 00
	Nitrogen	2.00*E* + 02	kg/yr	67.8	1.64	1.36*E* + 04	3.28*E* + 02
	Phosphate	1.10*E* + 02	kg/yr	36.2	1.05	3.98*E* + 03	1.16*E* + 02

Total						6.80*E* + 04	3.03*E* + 03

*Zhou [24].

**Table 3:** Direct GHG emissions of the “pig-biogas-fish” system.

Direct GHG	CO_2_ (kg/yr)	CH_4_ (kg/yr)	N_2_O (kg/yr)
Pigsty		12.00*	
Biogas	830.50**		1.14***
Fishpond****	646.88	45.36	

*IPCC [26].

**Biogas composition is considered as 70% CH_4_ and 30% CO_2_.

***Ma  and Nan [27].

****Xing et al. [28].

**Table 4:** GE of the “pig-biogas-fish” system.

	Direct GHG (kg CO_2_-eq/yr)	GE_in_ (kg CO_2_-eq/yr)	GE (kg CO_2_-eq/yr)
Pigsty	2.76*E* + 02	1.76*E* + 03	2.04*E* + 03
Biogas	1.17*E* + 03	3.16*E* + 02	1.48*E* + 03
Fishpond	1.69*E* + 03	9.56*E* + 02	2.65*E* + 03

Total	3.13*E* + 03	3.03*E* + 03	6.17*E* + 03
Ratio	50.82%	49.18%	100.00%

**Table 5:** FE cost and GE_in_ emissions of the conventional animal husbandry system.

	Materials	Quantity	Unit	*C*_i_* (MJ/unit)	*G*_i_* (kg CO_2_-eq/unit)	FE (MJ/yr)	GE_in_ (kg CO_2_-eq/yr)
Pigsty	Cement	1.75*E* + 01	kg/yr	6.36	0.53	1.11*E* + 02	9.28*E* + 00
	Lime	1.25*E* + 01	kg/yr	4.94	0.79	6.18*E* + 01	9.88*E* + 00
	Hollow bricks	1.50*E* + 00	kg/yr	6.36	0.53	9.54*E* + 00	7.95*E* − 01
	Steel	2.63*E* + 00	kg/yr	64.5	2.03	1.69*E* + 02	5.33*E* + 00
	Feed	2.90*E* + 03	kg/yr	4.64	0.52	1.35*E* + 04	1.51*E* + 03
	Drugs	4.80*E* − 02	kg/yr	134	3.00	6.43*E* + 00	1.44*E* − 01
	Disinfectant	4.00*E* − 01	kg/yr	46.9	1.68	1.88*E* + 01	6.72*E* − 01
	Water	5.66*E* + 01	ton/yr	34.6	0.81	1.96*E* + 03	4.58*E* + 01
	Electricity	6.40*E* + 01	kWh/yr	135	2.82	8.64*E* + 03	1.80*E* + 02

Farmers	Coal	2.86*E* + 02	kg/yr	29.56	3.2	8.44*E* + 03	9.14*E* + 02

Fishpond	Lime	4.80*E* + 02	kg/yr	4.94	0.78	2.37*E* + 03	3.74*E* + 02
	Bleach	3.20*E* + 00	kg/yr	46.9	1.68	1.50*E* + 02	5.38*E* + 00
	Feed	2.50*E* + 02	kg/yr	4.64	0.52	1.16*E* + 03	1.30*E* + 02
	Aerator	1.25*E* + 00	$/yr	32.4	2.24	4.05*E* + 01	2.80*E* + 00
	Nitrogen	2.00*E* + 03	kg/yr	67.8	1.64	1.36*E* + 05	3.28*E* + 03
	Phosphate	1.10*E* + 03	kg/yr	36.2	1.05	3.98*E* + 04	1.16*E* + 03

Total						2.12*E* + 05	7.62*E* + 03

*Zhou [24].

**Table 6:** GE of the conventional animal husbandry system.

	Direct GHG (kg CO_2_-eq/yr)	GE_in_ (kg CO_2_-eq/yr)	GE (kg CO_2_-eq/yr)
Pigsty	2.76*E* + 02	1.76*E* + 03	2.04*E* + 03
Farmers	0.00*E* + 00	9.14*E* + 02	9.14*E* + 02
Fishpond	1.69*E* + 03	4.95*E* + 03	6.64*E* + 03

Total	1.97*E* + 03	7.62*E* + 03	9.59*E* + 03
Ratio	20.50%	79.50%	100.00%
